# New algorithm for constructing area-based index with geographical heterogeneities and variable selection: An application to gastric cancer screening

**DOI:** 10.1038/srep26582

**Published:** 2016-05-24

**Authors:** Daisuke Yoneoka, Eiko Saito, Shinji Nakaoka

**Affiliations:** 1 Department of Statistical Science, School of Multidisciplinary Sciences, SOKENDAI (The Graduate University for Advanced Studies), 10-3 Midori-cho, Tachikawa, Tokyo 190-8562, Japan; 2 Graduate School of Medicine, The University of Tokyo, 7-3-1 Hongo, Bunkyo-ku, Tokyo 113-0033, Japan

## Abstract

To optimally allocate health resources, policy planners require an indicator reflecting the inequality. Currently, health inequalities are frequently measured by area-based indices. However, methodologies for constructing the indices have been hampered by two difficulties: 1) incorporating the geographical relationship into the model and 2) selecting appropriate variables from the high-dimensional census data. Here, we constructed a new area-based health coverage index using the geographical information and a variable selection procedure with the example of gastric cancer. We also characterized the geographical distribution of health inequality in Japan. To construct the index, we proposed a methodology of a geographically weighted logistic lasso model. We adopted a geographical kernel and selected the optimal bandwidth and the regularization parameters by a two-stage algorithm. Sensitivity was checked by correlation to several cancer mortalities/screening rates. Lastly, we mapped the current distribution of health inequality in Japan and detected unique predictors at sampled locations. The interquartile range of the index was 0.0001 to 0.354 (mean: 0.178, SD: 0.109). The selections from 91 candidate variables in Japanese census data showed regional heterogeneities (median number of selected variables: 29). Our index was more correlated to cancer mortalities/screening rates than previous index and revealed several geographical clusters with unique predictors.

The public health sector is concerned with not only individual health but also the health of different areas. Measuring the area health condition, especially the “health inequality”, is a critical aspect of policy making. Therefore, to optimally allocate health resources and services, health policy planners require indexes that properly quantify this inequality^1^. Moreover, a health related index should be based on easily accessible data. This is particularly important in Japan, whose population has the highest health status in the world^2^, because individual patient data (specifically those of cancer, the leading cause of death) are difficult to obtain^3^ and constructing the index under Japanese setting is beneficial to other countries.

As a proxy of health inequality, many health policy studies adopt the area-based health coverage index, which is based on administratively defined boundaries. For example, the allocation of additional medical resources to practitioners is decided by the Jarman underprivileged area score, and the Townsend Z-score has been widely used for measuring inequalities. A variant of the Townsend Z-score, the Corsairs index, incorporates the level of the individual^4^,^5^,^6^. Most indices are weighted combinations of area-based predictors such as employment status and car ownership, usually measured at municipality levels. These indices are simple summations of selected area-based variables. The logistic regression approach was first applied to index construction by Gordon *et al*. in^7^ for estimating the weightings of area-based variables^7^. On the basis of Gordon’s^7^ procedure, Nakaya^8^ adapted an index to a Japanese setting^8^. The composite variables selected by Nakaya^8^ were similar to those of the European transnational ecological measure^8^,^9^,^10^. The same regression framework is followed in the present study.

The above methodologies assume that current areas are homogeneous; i.e., they assign the same weights and variable sets to all independently sampled locations. Uniform assignment implies that the factors that explain the area inequality are identical at all locations, whereas independent sampling obscures any geographical correlation even if two areas are located in the same neighborhood. In realistic settings, the health inequality in single and neighboring areas should be geographically correlated, and individual areas should have different sets of predictors. Although the above methodologies are easily implemented, the unrealistic homogeneity assumption needs to be relaxed in practice. To this end, we must overcome the following difficulties: 1) incorporation of geographical information into the model and 2) appropriate variable selection from high-dimensional census data.

Geographical information can be integrated by a geographically weighted regression (GWR) model that incorporates spatial nonstationarity^11^,^12^. The GWR is a variant of a local regression model with a spatial weights kernel, in which the regression coefficients depend on the data point locations, and the kernel weights are estimated from the distances between data points^13^. The variable selection problem can be mitigated by a lasso (least-squares absolute shrinkage and selection operator) regularization. The lasso model provides good variable selection procedure, especially when the number of available variables in the local regression model is enough large than the number of sample areas because the least significant variable coefficients are shrunk toward zero. Compared with previous indices, whose variables are often arbitrarily selected on the basis of theoretical knowledge^7^, the lasso approach systematically selects the variables from the dataset. The data-driven property of lasso enables a reproducible result that is easily extrapolated to other data; For example, the lasso model has been used to find useful risk predictors among hundreds of gene data for lung or breast cancer^14^,^15^. The lasso model also alleviates the multicollinearity problem. Wheeler *et al*.^16^ showed that GWR coefficients can be systematically correlated even if the covariates are noncollinear, because collinearity among covariates is affected by the spatial kernel of the GWR. Such collinearity of locally weighted covariates can indicate strong dependency between the local coefficients^16^. Extending Wheeler’s^17^ procedure, this study introduces a geographically weighted logistic lasso regression into a statistical methodology for constructing an area-based health coverage index^16^. The aim is to propose how to construct a new health coverage index and to provide an example index for cancer screening.

## Methods

### Geographically weighted logistic regression (Logistic GWR)

The geographically weighted logistic regression (Logistic GWR) model can be defined in terms of a spatially varying coefficients model at each sampled location^17^,^18^,^19^. The spatially varying logistic regression model at sample location *i* (1, …, *N*) is given by


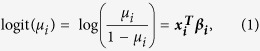


where *μ*_*i*_ = *E*(*y*_*i*_ = 1|***x***_***i***_), *y*_*i*_ ∈ {0, 1} is a binary outcome variable, ***x***_***i***_ = (1, *x*_*i*1_, … *x*_*ip*_)^*T*^ is a covariate vector, and ***β***_***i***_ is a (*p* + 1) × 1 vector of coefficients. Because this model has *N* samples and *N*(*p* + 1) coefficients, it is nonidentifiable. Therefore, to estimate valid regression parameters, we must strengthen the effects of the neighboring locations by incorporating weights^17^. For determining the spatial dependencies among the covariates, we calibrate the weights by the distances between the sample coordinates. Given a weight *w*_*ij*_ between any location *j* and a model calibration point *i*, the local log-likelihood at location *i* is calculated as


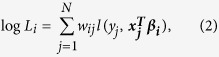


where *l*(*y, η*) is the negative log-likelihood contribution. The distance can be calculated in various ways; e.g., the Minkowski distance, which reduces to the Euclidean distance when *p* = 2 and the Manhattan distance when *p* = 1 (where *p* is the power of the Minkowski distance). Here, we adopt the usual Euclidian distance, and the Gaussian distance decay-based weighting function proposed by Brunsdon *et al*.^18^ as the weight kernel^18^. This function is defined as 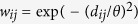, where *d*_*ij*_ is the distance between location *j* and model calibration point *i* and *θ* is the bandwidth parameter.

### Geographically weighted logistic lasso regression (Logistic GWL)

We now adapt the Logistic GWR to the lasso model. The resulting model, called geographically weighted logistic lasso regression (Logistic GWL), constrains the regression coefficients by adding a lasso penalty term to the local likelihood (2) as follows:


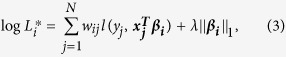


where the shrinkage parameter *λ* controls the overall strength of the *L*_1_ norm penalty 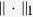. The parameters related to the shrinkage *λ* and the kernel bandwidth *θ* are estimated by leave-one-out cross-validation (LOO–CV), minimizing the prediction accuracy criteria 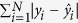. This method is extendible to more complex cases such as the ridge or the elastic net model by incorporating another type of penalty term^20^.

The inference algorithm proceeds in two steps: the first step decides the optimal bandwidth and the shrinkage parameter by LOO–CV; the second step decides the final Logistic GWL solution. The pseudocode of the first step is given in Algorithm 1.

**Algorithm 1**

1. Set a sequence of bandwidth candidates 
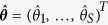
 and shrinkage parameters 
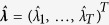
 and calculate the *n* × *n* distance matrix ***D***.
2. Repeat for each location *s* = 1:*S*;
  1. Calculate the *n* × *n* weight matrices ***W***_***s***_ from ***D*** and 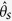 with a Gaussian kernel function. Row *i* and column *j* of ***W***_***s***_ contains the *w*_*ij*_ defined in Equation (3).
  2. Repeat for each location *t* = 1:*T*;
  - Repeat for each location *i* = 1:*N*;
    - Set *w*_*s*,*i*_ = *W*_*s*_[*i*, −*i*], an (*N* − 1) × 1 vector that removes the *i*th location from the weighting.
    - Maximize the penalized likelihood (Equation (3)) with the lasso penalty term 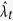 and save the lasso solution.
    - Test the model on the *i*th sample and save the prediction criteria.
3. Find the 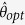 and 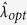 that minimize the prediction criteria.

In Algorithm 1, the lasso solutions at the optimal bandwidth 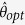 and 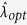 among 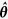 and 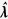 are found by a binary search technique.

The second step estimates the final Logistic GWL solution, as shown in Algorithm 2.

**Algorithm 2**

1. Calculate ***W*** from the estimated 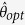 and ***D***.
2. Repeat for each location *i* = 1:*N*; 
  1. Maximize the likelihood (Equation (3)) with the penalized parameter 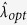 estimated in the first step.

The R package “GWLelast” is available on CRAN at http://cran.r-project.org/web/packages/GWLelast/index.html.

### Construction of the health coverage index and data explanation

Similar to Gordon^7^, the health coverage index was computed as 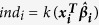, where *ind*_*i*_ refers to the index at location *i*^7^. The weightings in this expression were the estimated odds ratios of the Logistic GWL. The other parameters are 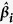, the *i*th estimated set of coefficients, which indicates the spatial variability among the coefficient sets, and ***x***_***i***_, the *i*th set of covariates remaining in the Logistic GWL. The adjustment constant *k* was proposed by Gordon^7^ to match the population average to the sum of the *ind*_*i*_ weighted by the number of households^7^.

The present study used the 91 variables in the 2010 Japanese census data. In 2010, there were 1743 municipalities in Japan, each consisting of 47 prefectures. Cancer screening rates and mortalities during 2010 were recorded by the Ministry of Health, Labour and Welfare of Japan.

The dependent variable in the Logistic GWL was the gastric cancer screening rate binarized by the sample median to apply the concept of the Gordon’s method. Although we temporary assumed that the sample median was a threshold to distinguish the gastric cancer rate, this method can be extended to any other values of threshold. To choice the optimal threshold value, we can propose several well-known methods such as cross-validation, the use of validation dataset and sensitivity analysis by changing the threshold value. Further, although the dependent variable was temporary assumed to be binary, this method can also be generalized to other type of dependent variables by changing the link function in generalized linear model (GLM) (Note that the R package “GWLelast” can use other regressions in GLM family such as poisson and probit regression). Previous studies have shown that the screening rate suitably represents the area-dependent health status because it measures the degree of the accessibility to preventive health services and also have shown that the health status depends on the geographical factors^21^,^22^,^23^. Therefore, our index should be considered to reflect the number of population members with low health status because cancer is the major cause of death in Japan^24^,^25^. Especially, the gastric cancer was important and a major disease in Japan with the second place in the mortality and the first place in the morbidity among all cancer, and thus it is necessary to construct the coverage index to predict the screening rate of gastric cancer. The 91 covariates in the Japanese census data are described in Supplemental dataset 1. All covariates were normalized beforehand. As a sensitivity analysis, our proposed index was compared with that of Nakaya^8^. The screening rates and mortalities of cancers other than gastric cancer (cervical, colon, breast, liver, and lung cancer) were also examined by correlation analyses based on Spearman’s correlation test.

The algorithm returns a *N* × (*p* + 1) sparse matrix of coefficients. To analyze the tendency of the sparseness (i.e., the number of nonzero coefficients in the sampled locations), the sparse matrix was processed by a co-clustering technique^26^. The co-clustering algorithm simultaneously clusters the rows and columns depending on the relationships between two entities of interest. It consists of a mixture model that estimates the block clusters on both individual and variables sets. This analysis detects the unique covariates in each area highly correlated with health coverage. The number of row and column clusters was equal and set to 10.

## Results

**Figure 1:**
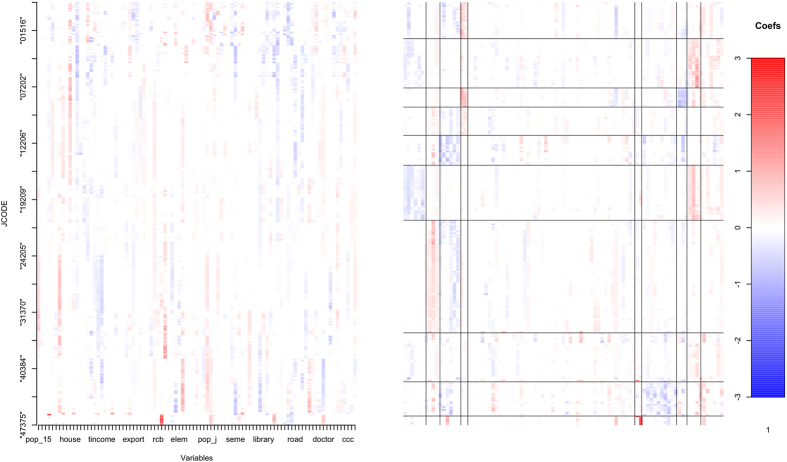
Heatmap of the estimated coefficient matrix (left) and the result of co-clustering of the coefficient matrix (right). All illustrations were created using the R software (v.3.1.1, http://www.r-project.org).

The first algorithm in the methods sections yielded an optimal bandwidth of 5.9 and an optimal shrinkage parameter of 0.0116. The estimated coefficients and the result of co-clustering are presented as the heatmap form in Fig. 1 (left: the matrix of estimated coefficients, right: the result of co-clustering of the coefficient matrix). The result shows each cluster has unique variable set and the selected variables vary across clusters, which enhances the flexibility of our method. More detailed values are reported in the Supplemental dataset 2.

**Figure 2:**
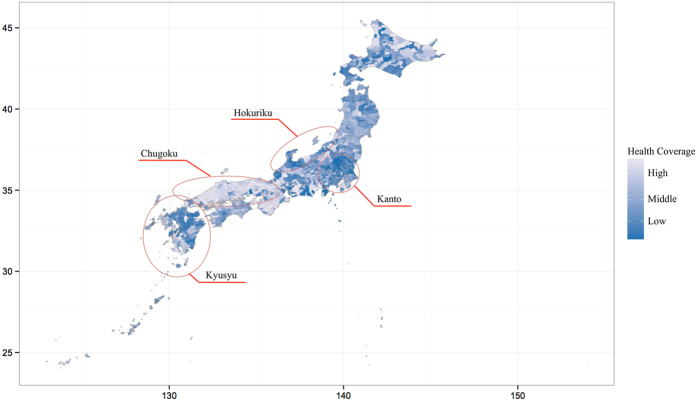
Mapping of area-based health coverage index in Japan. This illustration was created using the R software (v.3.1.1, http://www.r-project.org).

In this study, the population average of the screening rate of gastric cancer was 10.18%; therefore, *k* was set to 0.354. Figure 2 maps the estimated health coverage indexes across Japan. The index ranged from 0.0001 to 0.354 (mean: 0.178, median: 0.168, SD: 0.109). Areas of low coverage were clustered at the center of Kyusyu Island, the interior regions of Kanto and the Hokuriku region. On the other hand, the west part of Honsyu (Chugoku area) shows high coverage. The results of the application to other types of outcomes such as all cancer mortality are reported in the Supplemental dataset 3 and 4.

The average number of selected variables was 28 (median: 29, SD: 4.4; see Supplemental dataset 2 for details). The three most frequent covariates with nonzero coefficients were “fire,” denoting the proportion of fires per total population (1426 selections out of 1743 locations), “rer,” the ratio of net excess revenue in the municipality (1339 selections), and “house_n,” the proportion of nuclear families per total number of households (1275 selections). The top three contributing covariates (i.e., the three covariates with the largest absolute values of coefficients) were “house,” the proportion of households per total population (mean absolute value: 0.210), “movein,” the proportion of move-ins per total population (mean absolute value: 0.190), and “pop_j,” the labor force population among the total population (mean absolute value: 0.185). According to the co-clustering results of the coefficient matrix (the resultant heatmap is in Fig. 1 (right)), the Kyusyu area showed a characteristically high correlation between health coverage and specific covariates (namely, “juni_s”: the proportion of students in junior high schools per total population, “high_s”: the proportion of students in high schools per total population, and “semo,” the proportion of self-employed without employees among the employed population). In the Kanto and Hokuriku areas, coverage was characteristically correlated with “house_ac,” the proportion of aged-couple households per total number of households, and “bigretail,” the proportion of big retailers per total population, respectively.

Table 1 shows the results of the correlation analysis. The correlation between our proposed index constructed from the gastric cancer screening rate and Nakaya’s^8^ index was −0.181 (p < 0.001). Our index was also relatively highly correlated with the screening rates of other cancers; namely, cervical: 0.412 (p < 0.001), colon: 0.572 (p < 0.001), breast: 0.541 (p < 0.001), and lung: 0.516 (p < 0.001). Furthermore, our index was correlated with cancer mortality; all cancers: −0.335 (p < 0.001), gastric: −0.163 (p < 0.001), colon: −0.250 (p < 0.001), liver: −0.478 (p < 0.001), and lung cancer: −0.343 (p < 0.001).

## Discussion

We propose a general methodology and algorithm for constructing an area-based health coverage index using the geographically weighted logistic lasso approach. Our index was constructed from the census data at the municipality level. While previous area-based indices assume the same weights and variables across all municipalities and no correlation between sample locations, our proposed method allows a more flexible formulation with location-dependent weights and variables that accommodate the geographical relationships. In addition, due to the lasso property, our method automatically can select the relevant covariates from high-dimensional data such as census data without the arbitrariness of the selection of variables.

Our results indicate several clusters of low coverage areas with characteristic predictors, implying that the proposed method flexibly predicts the health inequality with unique covariates at each location and that the selected variables vary across clusters. The implications of these selected predictors require further epidemiological research. Our index was significantly correlated with the cancer screening rate and mortality of gastric cancer and relatively highly correlated with those of other cancers. In contrast, Nakaya’s^8^ index, which is frequently used as a covariate in Japanese cancer studies, is uncorrelated with these cancer variables^8^,^27^,^28^. Given its favorable properties, our index provides a potentially useful adjustment factor of health coverage in future studies.

Spatial statistical analysis has become a standard approach in epidemiological research^29^,^30^,^31^. Therefore, numerous methods applying different formulations are available for variable selection. For example, we could have adopted ridge regression or partial least-squares regression. However, the lasso is desirable because it ensures sparsity of coefficients and interpretability of the covariate effects by directly reducing the regression variance^17^. In addition, compared with existing methods for constructing area-based index (e.x. principal component analysis (PCA)), the lasso is a promising tool to select important variables because it does not have such disadvantage as follows; The dimension reduction methods such as PCA are not necessarily to be able to extract the common unique dimension for compositing the index and the weights in the composite index may not correspond to an importance of the variable^8^. On the other hand, as frequently reported in spatial epidemiology studies, the result is sensitive to the geographical unit^32^. Thus, we must evaluate and compare the validity of our proposed and previous indices in several geographical units (e.g., Krieger *et al*.^33^).

A major limitation of our method is its reference to aggregated data (in this case, census data) rather than individual data. As such, it requires cautious interpretation to avoid the well-known ecological fallacy, in which the results differ between the group and individual levels^34^. To overcome this limitation, multilevel analysis incorporating the data of both individuals and municipalities will be required^35^,^36^,^37^. Then, although the sparsity of the lasso model secures its effectiveness as a variable selection procedure, it is prone to several problems; e.g., the coefficient estimators are not statistically consistent and the variables are arbitrarily selected^20^,^26^,^38^. The lasso method tends to shrink the estimated coefficients toward zero to improve the prediction accuracy, leading to bias and lack of statistical consistency instead of reduced variance (known as the bias–variance tradeoff)^26^. Unbiased coefficients can only be obtained by a debiasing procedure such as re-calculation of the nonzero coefficients derived by the lasso^26^. Further, if any of the selected variables are highly correlated, it is generally preferable to select all relevant variables in the group, but the lasso model is likely to arbitrarily select only one of them. To solve this problem, our method can be easily extended to an elastic net model, a hybrid of lasso and ridge regression, by adding the *L*_2_ penalty of the coefficients to the penalty term in Equation (3)^20^. This option is available in our proposed R package “GWLelast”.

Japan will launch a national cancer registration system in 2016. Meanwhile or even after the launch, large-scale individual health data for predicting health coverage in cancer remain difficult to obtain^3^. Our study suggests that area-based data provide a suitable proxy of the inequality measure and could assist health policy making.

## Conclusions

We developed novel geographical model (and R package) for a area-based health coverage index including geographical information and a variable selection procedure. We also characterized health inequality of coverage in Japan and found several clusters with unique predictors. This model added flexibility of the geographical heterogeneities by a geographically weighted lasso logistic regression model, which showed stronger correlation for cancer prediction than a previous index.

## Additional Information

**How to cite this article**: Yoneoka, D. *et al*. New algorithm for constructing area-based index with geographical heterogeneities and variable selection: An application to gastric cancer screening. *Sci. Rep.* **6**, 26582; doi: 10.1038/srep26582 (2016).

## Supplementary Material

Supplementary Information

Supplementary Information

Supplementary Information

## Figures and Tables

**Table 1:** Correlation results of our index constructed from gastric cancer screening rate and Nakaya’s index.

	Proposed index		Nakaya	
	Correlation*	p-value**	Correlation	p-value
Nakaya’s Index				
	−0.181	<0.001	–	–
Cancer Screening				
Cervical	0.412	<0.001	−0.247	<0.001
Colon	0.572	<0.001	−0.224	<0.001
Breast	0.541	<0.001	−0.205	<0.001
Lung	0.516	<0.001	−0.201	<0.001
Cancer mortality				
All	−0.335	<0.001	−0.140	<0.001
Gastric	−0.163	<0.001	−0.284	<0.001
Colon	−0.250	<0.001	−0.137	<0.001
Liver	−0.478	<0.001	0.041	0.089
Lung	−0.343	<0.001	−0.023	0.340

^*^Sperman’s correlation.

^**^Null hypothesis is correlation = 0.
