# Supplementary material for: New algorithm for constructing area-based index with geographical heterogeneities and variable selection: An application to gastric cancer screening
Source: Sci Rep. 2016 May 24;6:26582. doi: 10.1038/srep26582 (PMC4877577; doi:10.1038/srep26582)

New algorithm for constructing area-based index with geographical heterogeneities and variable selection: An application to gastric cancer screening

Daisuke Yoneoka*, Eiko Saito **, Shinji Nakaoka ***

List of Supplemental datasets:

Supplemental dataset 1: Variable list from 2010 census data.

Supplemental dataset 2: Table that illustrates the estimated coefficients and the deprivation index.

filetype: xls (in Extra file)

Supplemental dataset 3: Mapping of area-based health coverage index created from other examples in Japan. This illustration was created using the R software (v.3.1.1, http://www.r-project.org).

Supplemental dataset 4: Table that illustrates the deprivation index constructed from other examples.

filetype: xls (in Extra file)

Supplemental Table 1: Variable list from 2010 census data

Var name	Descriptions	Var name	Descriptions	Var name	Descriptions
pop_15	Porportion of population under 15 year per total populations	emp_c	Proportion of populations employed in commercial enterprises per total populations	wpe	Proportion of workers by place of employment per total employments
pop_15to64	Porportion of population between 15-64 year per total populations	fi	Financial capability index	com_from	Proportion of persons commuting from other towns per total employments
pop_65	Porportion of population over 65 year per total populations	rer	Ratio of net excess revenue	comhall	Proportion of community halls per total populations
pop_for	Proportion of foreigners per total populations	rcb	Ratio of net cost of bonds	library	Proportion of libraries per total populations
birth	Proportion of birth per total population	revenue	Proportion of revenue of the municiparity per total populations	pwosl	Proportion of populations without sewage line per total populations

death	Proportion of death per total population	expend	Proportion of expenditure of the municiparity per total populations	pop_g	Proportion of population involving garbage collection as a job per total populations
movein	Proportion of move-ins per total population	regtax	Proportion of regional tax of the municiparity per total populations	garbage	Proportion of total garbage tonnage per total populations
moveout	Proportion of move-outs per total population	kinder	Proportion of kindergardens per total populations	recyrate	Recycle rate
pop_day	Proportion of daytime population per total population	chil_k	Proportion of children in kindergardens per total populations	retail	Proportion of retail shops per total populations
house	Proportion of huoseholds per total population	elem	Proportion of elementary schools per total students in elementary schools	rest	Proportion of restaurants per total populations
house_p	Proportion of private households per total households	elem_t	Proportion of techers in elementary schools per total students in elementary schools	bigretail	Proportion of big retaillers per total populations

house_n	Proportion of nuclear families per total households	elem_s	Proportion of students in elementary schools per total populations	department	Proportion of department store per total populations
house_o	Proportion of one-person families per total households	juni	Proportion of junior high schools per total students in junior high schools	road	Proportion of road (km) per total area
house_n65	Proportion of nuclear families with relatives over 65 yr per total households	juni_t	Proportion of techers in junior high schools per total students in junior high schools	road_m	Proportion of main road (km) per total area
house_ac	Proportion of aged-couple households per total households	juni_s	Proportion of students in junior high schools per total populations	road_mu	Proportion ofl municipal road (km) per total area
house_as	Proportion of aged-single households per total households	high	Proportion of high schools per total students in high schools	road_p	Proportion of pavedl road (km) per total area
marriage	Proportion of marriages per total populations	high_s	Proportion of students in high schools per total populations	post	Proportion of post offices per total populations

divorce	Proportion of divorces per total populations	pop_j	Proportion of labor force population per total populations	hosp	Proportion of general hospitals per total populations
tincome	Proportion of taxable incomes per total taxpayes	emp	Proportion of employments per total labor force population	clinic	Proportion of general clinics per total populations
taxpayer	Proportion of taxpayers per total populations	uemp	Proportion of unemployments per total labor force population	dental	Proportion of dental clinics per total populations
ent	Proportion of enterprises per total populations	emp_p	Proportion of employments in primary industry per total employments	doctor	Proportion of doctors per total populations
ent_s	Proportion of secondary industry enterprises per total enterprises	emp_s	Proportion of employments in secondary industry per total employments	dentist	Proportion of dentists per total populations
ent_t	Proportion of tertiary industry enterprises per total enterprises	emp_t	Proportion of employments in tertiary industry per total employments	pharmac	Proportion of pharmacists per total populations

worker	Proportion of workers per total populations	empl	Proportion of employers per total employments	wccc	Proportion of waitlisted children for childcare centers per total populations
worker_s	Proportion of workersin secondary industry per total workers	board	Proportion of board menbers per total employments	nurhouse	Proportion of nursing houses per total populations
worker_t	Proportion of workersin tertiary industry per total workers	seme	Proportion of self-employed with employees per total employments	fdp	Proportion of facilities for disabled people per total populations
arable	Proportion of arable area per total area	semo	Proportion of self-employed without employees per total employments	childcare	Proportion of childcare centers per total populations
export	Proportion of product exports per total toal populations employed in maufacturing	fwork	Proportion of family workers per total employments	ccc	Proportion of children in childcare centers per total populations
emp_m	Proportion of populations employed in maufacturing per total populations	work_in	Proportion of persons working in their home town per total	nhir	Proportion of national health insurance reveivers per total

			employments		populations
sales	Proportion of annual sales per total populations employed in commercial enterprises	com_to	Proportion of persons commuting to other towns per total employments	fire	Proportion of fires per total populations
ent_c	Proportion of commercial enterprises per total populations				

Supplemental dataset 3: Mapping of area-based health coverage index created from other examples in Japan. This illustration was created using the R software (v.3.1.1, http://www.r-project.org).

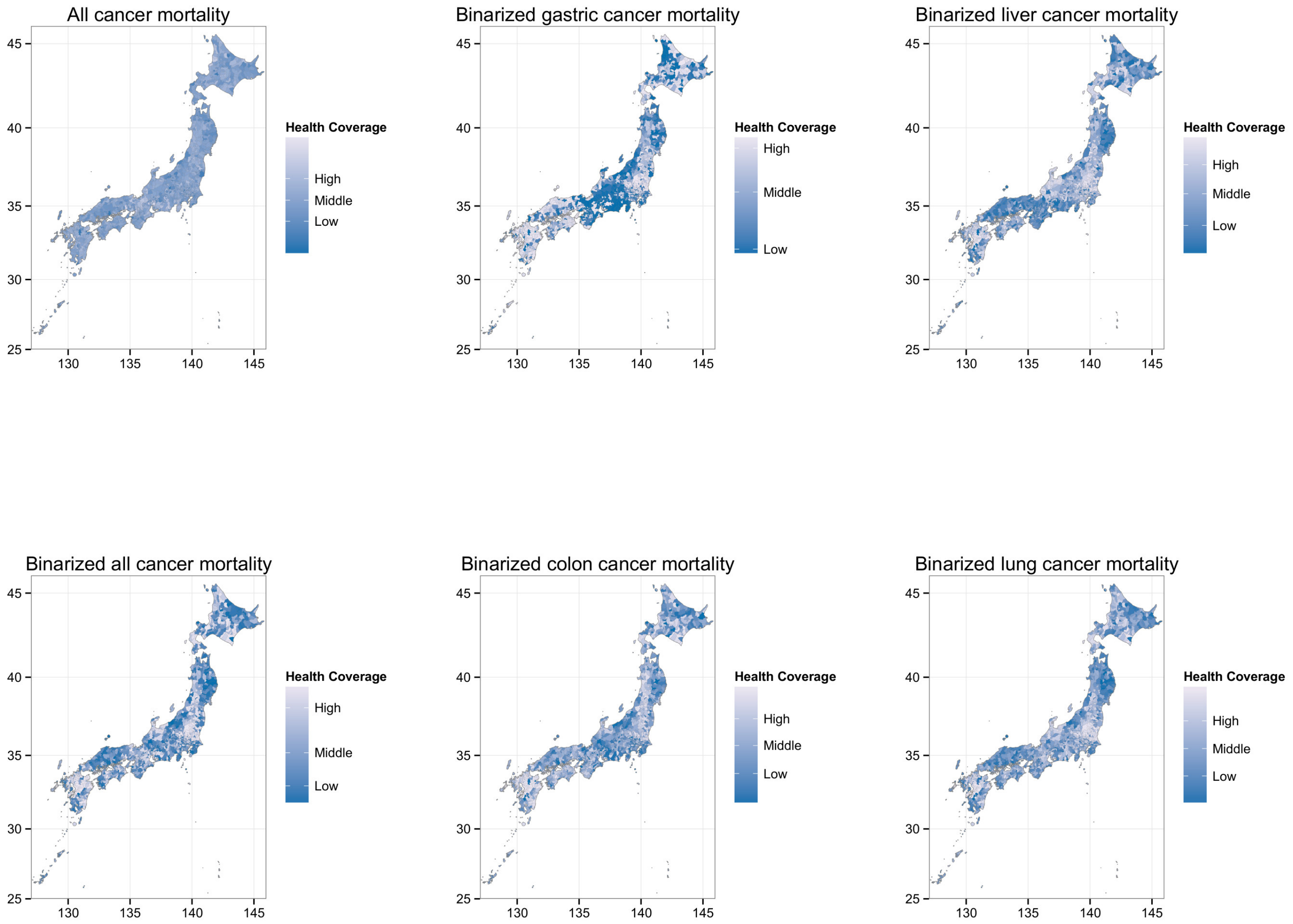

Supplement: Supplementary Information [file srep26582-s1.pdf]
